# Recent Advances on Chitosan-Based Nanoparticles for Brain Drug Delivery

**DOI:** 10.3390/polym17223055

**Published:** 2025-11-18

**Authors:** Chihab Ezzaki, Anas Chaari, Amani Al-Othman

**Affiliations:** 1 Biomedical Engineering Graduate Program, College of Engineering, American University of Sharjah, Sharjah P.O. Box 26666, United Arab Emirates; b00104672@aus.edu (C.E.);; 2 Biosciences and Bioengineering PhD Program, College of Engineering, American University of Sharjah, Sharjah P.O. Box 26666, United Arab Emirates; 3 Department of Chemical and Biological Engineering, College of Engineering, American University of Sharjah, Sharjah P.O. Box 26666, United Arab Emirates

**Keywords:** chitosan nanoparticles (CNPs), brain drug delivery, neuro-degenerative disorders, brain tumor, intranasal route

## Abstract

The blood–brain barrier (BBB) represents a major challenge in effective drug delivery systems intended for treating neurological disorders. It restricts the transport of therapeutic agents to the brain. Chitosan-based nanoparticles (CNPs) can be used for brain drug delivery because of their biocompatibility, biodegradability, and ability to enhance drug permeability across the BBB. This review article discusses the design and application of CNPs for brain-targeted drug delivery, exploring their mechanisms of action, including adsorptive-mediated and receptor-mediated endocytosis. Surface modifications with ligands such as chlorotoxin are discussed for improving specificity and therapeutic results. Findings show that CNPs allow controlled drug release, enhance stability, and reduce side effects, which make them effective for treating multiple neurological conditions, including Alzheimer’s disease, Parkinson’s disease, brain tumors, and ischemic stroke. CNPs can encapsulate multiple therapeutic agents, such as anti-inflammatory drugs, cytotoxic agents, and genetic materials, and maintain stability under different physiological conditions. Intranasal delivery routes are mainly discussed in this paper for their ability to bypass systemic circulation and achieve direct brain targeting. This review also addresses challenges such as cytotoxicity and the need for optimizing nanoparticle size, charge, and surface properties to improve the therapy results. While CNPs are suitable for brain drug delivery, there is a research gap, which is the lack of systematic studies evaluating their long-term effects on brain tissue and health. Most studies focus on acute therapeutic outcomes and in vitro or short-term in vivo analysis, which do not address some questions about the chronic exposure risks, biodistribution, and clearance pathways of CNPs. This review also explores the use of chitosan-based nanoparticles to deliver drugs to the brain for the treatment of multiple neurological disorders.

## 1. Introduction

Brain drug delivery represents an interesting topic that is accompanied by multiple challenges in the field of drug delivery. The blood–brain barrier (BBB) is the inner wall of blood vessels in the brain that separates the blood stream from the brain and acts as a barrier that prevents the transportation of many microorganisms, chemicals, and neurotoxins to the brain for protection. As much as BBB is essential for protecting the brain, it represents an obstacle that restricts the transportation of drugs to the brain for treating certain neurological disorders and central nervous system (CNS) abnormalities.

The purpose of this review is to highlight the potential of chitosan-based nanoparticles in overcoming issues regarding effective brain drug delivery. Around the world, about 1.5 billion people are suffering from neurological disorders, such as Parkinson’s disease, Alzheimer’s disease (AD), multiple sclerosis, and many more [1]. This huge population imposes a necessity of directing efforts towards exploring methods of delivering drugs effectively to the brain for curing multiple brain diseases. The rise in these diseases is correlated with different factors such as aging, stress, and lifestyle. Another major challenge concerning brain drug delivery efficiency is the specific targeting of regions of brain disorders [2].

Nanoparticles have emerged as a technique with many potential applications for region-specific drug delivery due to their favorable properties, in addition to being efficient vehicles for transporting the drug across the BBB with effective doses; recent years have shown an increase in the utilization of nanoparticles for brain drug delivery [3]. Despite the exploration of different nanoparticles for drug delivery, only a few of them can actually deliver therapeutic molecules to damaged brain regions. Injection of a drug that is not encapsulated causes random distribution of the drug in the brain tissue, leading to multiple adverse consequences. Another problem is that achieving an unencapsulated drug concentration in the brain requires administering high doses, which can result in serious negative effects on surrounding normal tissue or drug toxicity. Therefore, drug degradation during delivery can be prevented by encapsulating them with nanoparticles that generally can adsorb a broad range of different drugs. This allows them to control the release of the drug, which provides a safe delivery method in case of delivering cytotoxic drugs to brain tumors with a suitable dose, and it prevents harming the normal healthy tissue. It also allows them to target specific brain regions and cross barriers to infiltrate pathways that are not accessible for free drugs (unencapsulated). Nanoparticles improve the stability of the drugs and extend the drug’s residence time in the body by controlling its release [4]. Chitosan-based nanoparticles (CNPs) can improve drug permeability by affecting the tight junctions across the BBB. Their ability to cross the BBB, as well as their biocompatibility since they are naturally derived polymers, makes them suitable for targeted drug delivery in neurological disorders and brain tumors. With surface modification, CNPs can carry tumor-targeting peptides such as transferrin and chlorotoxin, which increase their selectivity and specificity to brain tumors. Consequently, this minimizes the effect on healthy tissue and maximizes the effectiveness of treatment [5].

According to a study of CNPs by Khezri et al. [6] on brain tissue of rats, the chitosan nanocarrier (of around 79.5 nm) test group was able to increase “Zolmitriptan” concentration in brain tissue compared to the control groups for prolonged durations. CNPs have also exhibited drug protection and facilitation of drug transport across the BBB. This was due to the cationic (positively charged) nature of CNPs that allowed for adhesion to the negatively charged nasal mucosa in the nasal route; that, in turn, facilitated drug absorption and then transport to the brain, as the BBB is also a negatively charged membrane. The transport was further accelerated since the cells at the BBB are also negatively charged; the electrostatic interaction between the positively charged CNPs and cells is referred to as adsorptive-mediated endocytosis. Furthermore, CNPs were able to sustain their stability during different storage conditions for 6 months, which shows excellent encapsulation that prevents drug release.

CNPs are versatile in terms of surface modification; their surfaces can be easily modified to attach to specific ligands or molecules such as cholorotoxin, for example, as mentioned, which can specifically target brain tumors. These surface modifications are essential for a targeted delivery of certain regions in the brain and improving treatment results for various brain tissue disorders [7]. Nevertheless, a modified chitosan can be used in the coating of other nanoparticle polymers, such as Poly(lactic-co-glycolic acid) (PLGA) and Polylactic Acid (PLA), to improve BBB penetration, cell compatibility, and drug stability. Moreover, chitosan coating has been implemented for several nanocarriers such as micelles, iron oxide NPs, protein (lactoferrin) NPs, lipid carriers, N-isopropyl acrylamide (polymer) NP, etc. [8]. For these nanocarriers, it was reported that the chitosan coating provided enhanced cellular uptake, allocated surface modification for specific targeting, protection from enzyme degradation, controlled release, and biocompatibility to these nanocarriers. The less toxic, simple, stable, biocompatible, and completely biodegradable nature of chitosan, as well as BBB epithelial surface adhesion, gives it advantageous properties over other nanocarriers for treating neuro-degenerative diseases [9]. Other nanoparticles, such as polymeric miscelles, have poor cellular binding and uptake, which is essential for transportation through the BBB, and they also have a low drug loading capacity. Also, liposomal drug delivery systems are unstable as they often suffer from poor solubility, which leads to shorter circulation times. PLGA (unmodified) nanoparticles have also shown poor cellular uptake and difficulty in crossing the BBB due to their negative charge [10].

An oral route for brain drug delivery in terms of patient’s convenience; however, it has low brain targeting efficiency because of the BBB that limits the amount of drug reaching the brain [11]. Similarly, the BBB represents an obstacle that restricts drug concentration when it is delivered through the intravenous route, as well as the possibility of having systemic side effects [12]. A better alternative route for an effective delivery into the BBB is the intranasal route (through the nose), as it prevents the first-pass metabolism that can reduce the drug dose reaching the brain due to the high accessibility and great surface area of the olfactory cavity, making it a more direct transport route to the brain. The following table compares the oral, intravenous, and intranasal routes. Nevertheless, the muco-adhesive property of chitosan, since it is cationic, allows for prolonged residence time in the negatively charged nasal cavity, which eventually leads to enhanced drug absorption and effectiveness, allowing it to be more efficient in targeting specific brain regions [13]. For chitosan-based nanoparticles, their size should not exceed 300 nm for intranasal drug administration because higher dimensions minimize their ability for mucosal penetration and consequently prevent them from interacting with the underlying epithelial cell. According to the literature [14], nanoparticles smaller than 300 nm were easily transported through olfactory (nasal) neurons to the brain. Table 1 below shows comparisons between the intranasal, oral, and intravenous routes.

This paper fills a gap in the literature by addressing the use of chitosan for brain drug delivery via investigating the different routes and evaluating them in terms of targeting, bioavailability, and drug delivery effectiveness, which are crucial aspects for treating several serious brain conditions, such as cancer, neuro-degenerative diseases, and strokes, as a few examples. The article also discusses the factors contributing to the cytotoxic effects of the CNPs and how to minimize them. Despite the various review papers that were reported in the literature about brain drug delivery, there are still very few articles that demonstrate a comprehensive review that highlights the use of chitosan for brain drug delivery for different brain conditions. Given the promising properties of chitosan, it is aimed to attract the interest of researchers in this field to investigate optimal and effective techniques that can maximize disease treatment and minimize cytotoxicity.

## 2. Mechanisms of Brain Delivery with CNPs

**Figure 1:**
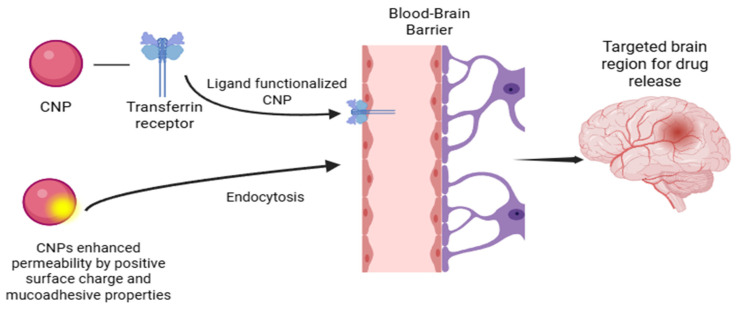
CNP applications: the concept of BBB penetration for brain drug delivery.

The brain–blood barrier “BBB” is constituted from adjoining layers of epithelial cells that have intricate tight junctions. The endothelial cells are specialized cells that line the internal surface of the BBB [15] and maintain the integrity of the BBB. The BBB complex comprises endothelial cells with embedded enzymes, transport channels, and receptors, which control the movement of ions, oxygen, nutrients, and other substances into the brain [16]. The crossing of nanoparticles for brain drug delivery for treating a certain disease is demonstrated in Figure 1.

As mentioned earlier, the delivery of CNPs through the intranasal route demonstrates improved brain drug delivery. According to a study made by Wei et al., intranasal administration of cyclovirobuxine D CNPs has shown highly enhanced brain availability for improving cognition and effective brain targeting [7]. After the intranasal administration, CNPs undergo endocytosis (i.e., uptake of nanoparticles into the bloodstream, where they are engulfed by endothelial cells, and vesicles are formed to internalize the nanoparticles), followed by transcytosis, where the endocytic vesicles carrying the administered nanoparticles transport them to the opposite side across the BBB and into the brain. These two transport phases across the BBB are affected by multiple factors such as size, shape, surface charge, and surface modification [17,18,19]. There is more than one method for the endocytosis of CNPs to occur, one of which is adsorptive-mediated endocytosis. Because of the cationic nature and muco-adhesive properties of chitosan due to the presence of amino groups, it interacts with the negatively charged endothelial cells at the cell membrane and opens the epithelial tight junctions. This leads to the transport of the CNPs into the blood and then them crossing into the brain tissue [20]. The adsorption of CNPs is also dependent on their degree of deacetylation; the lower the degree of deacetylation (DD), the greater their hydrophobicity, which improves their absorption since the cell membrane at the BBB is hydrophobic.

Given the factors affecting the transport of CNPs through the BBB (size, shape, surface charge/zeta potential), their evaluation can be explained by how the CNPs are manufactured. Manufacturing CNPs undergoes two processes: derivation of chitosan from isolated chitin (from aquaculture waste, crab shells) through deacetylation [21], and preparation of chitosan into stable, nanoscale particles, which can be performed through various techniques [22] such as ionic gelation, emulsion cross-linking, reverse micellar method, chemical modification, and many more [23]. Yang and Hon [22] have reported that smaller sizes for CNPs resulted from a higher degree of deacetylation (DD) (90%) (mean ~95 nm unloaded, ~113 nm with 5-Fluorouracil (5-FU) drug) compared to lower DD (75%) (mean ~176 nm unloaded, ~242 nm with 5-FU drug). Moreover, higher DD resulted in a more spherical shape, and higher surface charge area/zeta potential (+49.9 mV for 90% DD and +40.6 mV for 75% DD). A specific preparation technique can also impact the passage of CNPs through the brain–blood barrier (BBB), for example, ionic gelation yields positively charged particles (+25 to +54 mV as reported) in a size range of 300–400 nm based on chitosan/ Tripolyphosphate (TPP) ratio. Moreover, ionic gelation using TPP introduces zero cytotoxicity. Another example, for emulsion cross-linking, it mainly uses glutaraldehyde for chemical crossing [23]. With the use of Span-80, an emulsifier, Li et al. [24] have reported a particle size range of 100–300 nm depending on emulsifier content, content of cross-linking agent (glutaraldehyde), and oil to water volume ratio.

Another way is receptor-mediated endocytosis, an approach where the surface drug nanocarrier is modified with a ligand to selectively bind with a specific receptor at the BBB, which allows for precise delivery [16]. Multiple recent studies have shown that modified chitosan-based nanoparticles have repeatedly shown their superiority in brain drug delivery compared to unmodified ones in terms of specific targeting, stability, and/or BBB penetration. They can be modified by integrating molecules such as antibodies, peptides, or lipids. For example, CNPs conjugated with monoclonal antibody OX26 have shown improved BBB penetration. This antibody specifically targets the Transferrin Receptor (TfR) on the endothelial cell wall. The antibody-conjugated CNPs have shown more effective brain absorption of the drug compared to those that relied on adsorption for endocytosis, according to in vivo experiments performed on Balb/c mice. A lipid modification, such as grafting stearic acid into chitosan, improves BBB penetration, effective brain targeting, and extended drug release in vitro [16]. Moreover, Gupta et al. [5] mention that for specific targeting of brain glioma/tumors, the surface of CNPs is mainly modified with peptides such as transferrin and chlorotoxin as they have an increased selectivity for brain tumor cells; subsequently administering the carried drug to the cancer cells with minimized harm to the surrounding healthy tissue.

**Figure 2:**
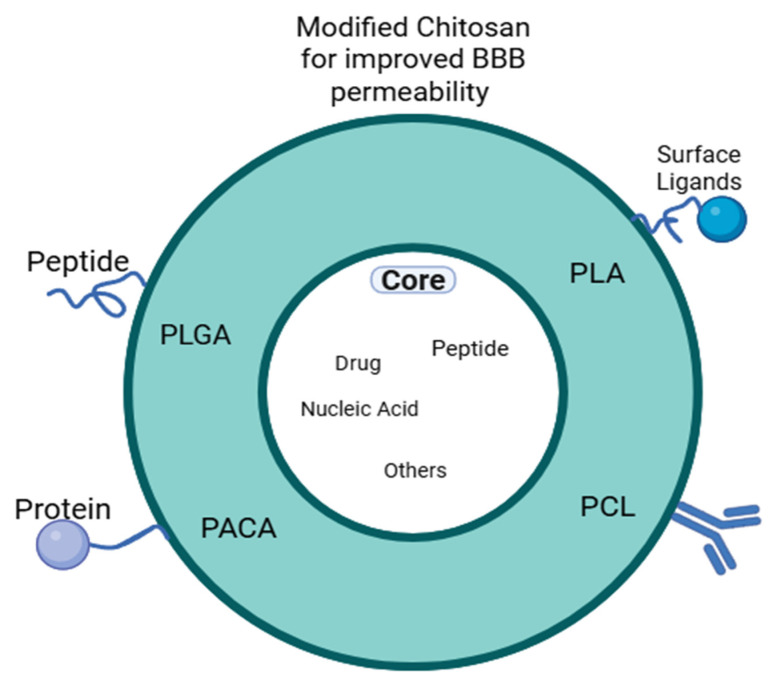
Different surface modifications that can be applied to CNPs for enhanced brain drug delivery.

Other surface modifications, such as coating with surfactants (Tween 80 or poloxamer), can improve the interaction with the endothelial cells at the BBB. Coating CNPs with polyethylene glycol stabilizes them and increases serum half-life. As shown in the previous examples, surface modification has many roles in improving BBB penetration, specific targeting, and drug absorption by the brain [7]. Figure 2 shows how different surface modifications can enhance the BBB penetration for nano-DDs (nano-drug delivery systems).

The release mechanism of CNPs to drugs is sustained as it undergoes two phases: initially, there is a rapid release of the drug, then it is followed by a continual steady release. Jiang et al. [25] have conducted a study in which they have synthesized and characterized cholic acid-loaded CNPs; then they observed the drug release in an in vitro assay over a time span of 24 h. The results showed a 20.99% drug release at first 30 min, then a 39.1% release at 6 h, 48.48% release at 12 h, and 49.1% release at 24 h. Authors have mentioned after listing the results how other studies on chitosan nanoparticles have demonstrated the same biphasic release profile of the drug, in addition to how continuous slow drug release over 24 h is highly advantageous as it prevents drug level variations, minimizes drug dosing frequency, and improves the efficiency of treatment. An increased chitosan polymer chain packing density of the nanoparticle decreases the rate of drug release, as well as larger sizes compared to smaller sizes [26].

In summary, this section discussed the mechanisms that allow chitosan nanoparticles to penetrate the BBB. The functions of adsorptive-mediated and receptor-mediated endocytosis are explained, and the effect of the physicochemical properties of CNPs, such as size, charge, and surface modifications, on their brain delivery efficiency has been discussed. This discussion shows the importance of rational design in the development of CNPs to produce optimal therapeutic outcomes in the brain.

## 3. Chitosan-Based Nanoparticles: Physical Properties, Preparation Methods and Factors Governing Interpolyelectrolyte Complexes for Brain Applications

The size and physicochemical properties of CNPs are important parameters for their efficiency in brain drug delivery applications. Several studies studied the optimal size range for crossing the blood–brain barrier, showing that CNPs between 30 and 400 nm are particularly effective. This size range facilitates entry into the brain by navigating through leaky barriers or inhibiting efflux pump activity, thereby enhancing BBB permeability [27]. For example, CNPs with a size of approximately 260 nm and developed using a complex coacervation method demonstrated efficient BBB crossing for in vivo mouse models, showing their ability for targeted brain therapies [28].

Other than size, the mechanisms of entry for CNPs into the brain are also influenced by their surface charge and functionalization. Nanoparticles conjugated with antibodies targeting the human TfR have shown increased cellular entry by receptor-mediated endocytosis, indicating the importance of ligand attachment in enhancing their therapeutic efficacy [29]. Additionally, low-molecular-weight chitosan nanoparticles have been shown to improve nanoparticle stability and facilitate BBB crossing [30]. In chitosan-based DDS, surface modification can be performed by covalent or non-covalent bonding. Non-covalent approaches use forces such as hydrogen bonding for better attachment to chitosan through functional group interactions, which enhance NP surfaces. Covalent modifications involve chemical reactions to link chitosan to NPs under conditions that maintain structural stability. For example, crosslinking with the carbodiimide reaction activates carboxyl groups that can then bond with amino groups on chitosan [16].

The zeta potential of Chitosan-based drug delivery systems is another major factor that determines their stability, cellular uptake, and effectiveness. Chitosan nanoparticles usually have a positive zeta potential because of the chitosan’s cationic nature, which enhances permeability across the blood–brain barrier by affecting tight junctions [31,32]. This positive charge can be modified by increasing chitosan concentration, which increases the electrostatic repulsion between particles, thus increasing zeta potential and stabilizing the colloidal system, or by modifying lipid content, which decreases the zeta potential to provide flexibility in formulation [33,34].

Furthermore, the zeta potential of chitosan nanoparticles changes with pH, as changes in protonation of amino groups affect solubility and drug retention, which makes it better for targeted delivery applications [35]. Drug loading to chitosan can reduce zeta potential because of the interactions between the drug molecules and the chitosan, which leads to an altered surface charge of the nanoparticle [36]. Therefore, enhanced chitosan nanoparticles with a stable, positive zeta potential, low PDI, and stability are suitable for achieving efficient therapeutic release.

CNPs are engineered using techniques such as ionic gelation and complex coacervation to produce nanoparticles around 260 nm in size that are suitable for crossing the BBB [28]. Chitosan can form polyelectrolyte complexes with other biopolymers, such as pectin, useful for drug encapsulation and controlled release [37]. López et al. [38] showed that chitosan-based nanogels can cross the BBB to deliver drugs effectively to brain regions, as shown through fluorescence microscopy in animal models. Surface modification of chitosan nanoparticles is an effective strategy to enhance BBB penetration and improve drug delivery efficiency. Techniques include incorporating chitosan-collagen nanocomposites and integrating other nanocarrier materials such as poly(lactic-co-glycolic acid) (PLGA) and poly(lactic acid) (PLA), which contribute to better targeting and drug release [9]. Another polycomplex that can be formed with chitosan is through PEGylation, in which PEG or polyethylene glycol is incorporated with chitosan. It increases the size of the chitosan nanoparticles and reduces the zeta potential or surface charge. Moreover, it contributes to the stability of CNPs by steric hindrance as it shields the positively charged chitosan surface with flexible hydrophilic chains, which reduces protein adsorption and opsonization and decreases interparticle attractive forces and aggregation. In addition, it improves blood/serum stability and improves biocompatibility [9,39].

A study performed by Rahman and Khalil [40] on PEGylated chitosan nanoparticles has shown an increased mean diameter of the chitosan nanoparticle from 143.7 nm to 175.6 nm and a decreased zeta potential from +25.7 mV to 22.9 mV. In addition, it enhanced the drug loading efficiency of the chitosan nanoparticles from 45.96% to 94.41%. Table 1 summarizes 14 studies showing different characteristics of chitosan-based nanoparticles for brain drug delivery. Particle sizes range from 16.12 nm to 255 nm, with most formulations under 200 nm optimal for crossing the blood–brain barrier. Positively charged nanoparticles (e.g., +32 mV) improve cellular uptake through electrostatic interactions with negatively charged membranes, improving bioavailability and therapeutic efficiency. Entrapment efficiencies change with surface modifications such as PEGylation, ligand conjugation (e.g., transferrin, chlorotoxin), and chitosan derivatives, improving mucoadhesion, stability, and receptor-mediated BBB transport. Table 2 shows a comparative analysis of chitosan-based nanoparticles for brain drug delivery.

Intranasal delivery is most used because it is non-invasive, and it provides direct CNS access, as seen in studies [43,47,54], which reported enhanced brain permeability and bioavailability. Intravenous routes used in studies such as [46,52] show systemic applications with ligand-targeted precision.

**Figure 3:**
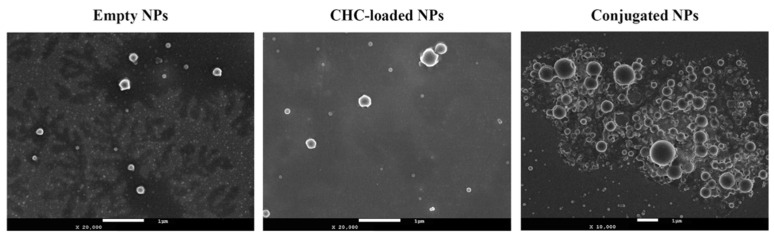
SEM photomicrographs of surface morphology of empty CNPs, CHC-loaded CNPs, and conjugated CNPs [42].

In the study made by Ferreira et al. [42], they aimed to design and optimize mucoadhesive PLGA–oligomeric chitosan nanoparticles (PLGA/OCS NP) for the delivery of both CHC (α-cyano-4-hydroxycinnamic acid) and cetuximab (CTX) drugs through the nasal-to-brain route for glioblastoma treatment. Eventually they were able to optimize these nanoparticles to have the following parameters: ~258 nm, PDI ~0.44 (polydispersity index, ranges from 0 to 1, closer to o means more uniform particle size), +37 mV zeta and ~88% CHC entrapment, which have led to a stable, nose-to-brain–relevant delivery system that has an efficient drug loading. Figure 3 below shows how the size of CNPs becomes bigger and more heterogeneous as they are loaded with the drug and conjugated with CTX. As seen, they are all, in general, spherical in shape. The empty NPs demonstrated larger spheres than the CHC-loaded NPs. Larger particles were observed for the conjugated but with a broad size distribution range.

**Figure 4:**
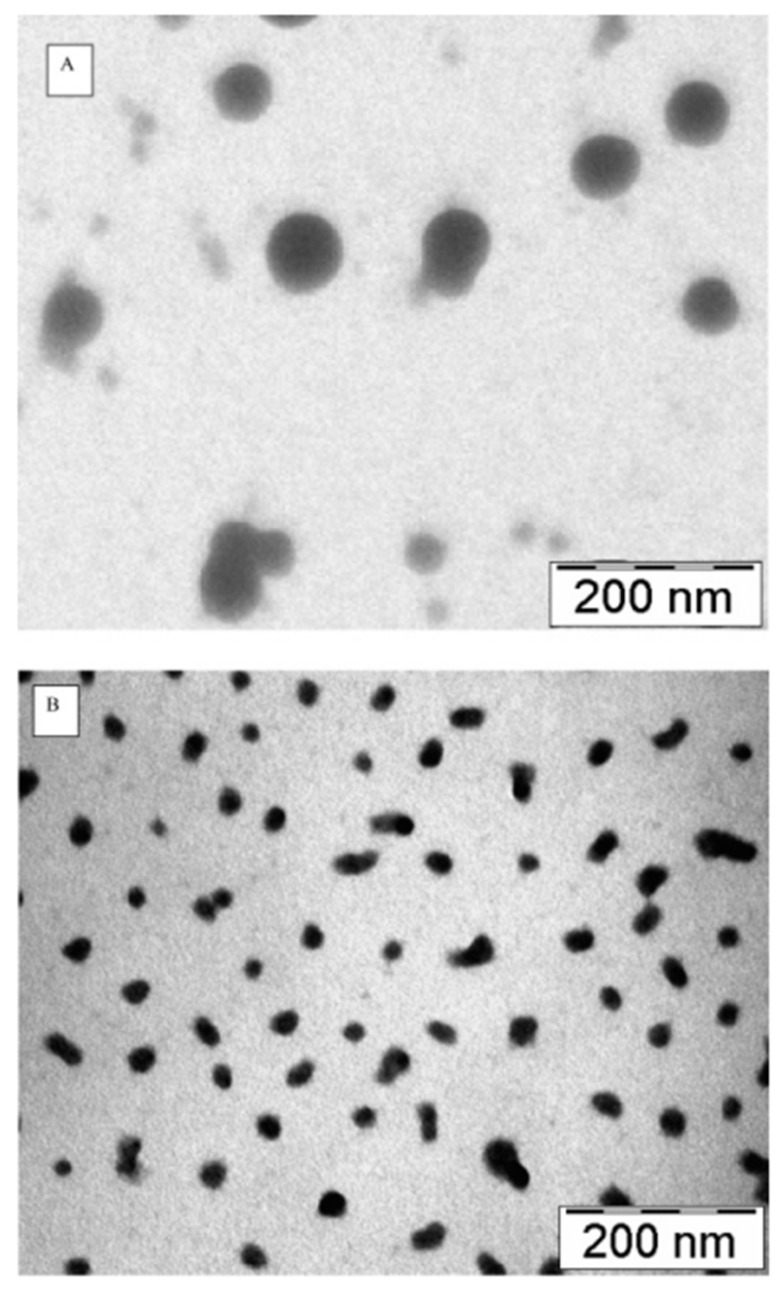
Transmission electron micrographs (TEM) of photographs of blank (**A**) CS nanoparticles; (**B**) PEG-CS nanoparticles [53].

In another study performed by Aktas et al. [53], they developed PEGylated CNPs that are functionalized with anti-transferrin receptor antibody OX26 to deliver caspase-3 inhibitor peptide through intravenous injection. The size of non-loaded PEGylated CNPs was approximately 150 nm, which is smaller compared to non-loaded unmodified CNPs (~339 nm). Figure 4 demonstrates the size change in CNPs before and after PEGylation via transmission electron (TEM) micrographs. Although PEGylation was made for the purpose of reducing the size of CNPs, stabilizing them, and providing a biotin handle for antibody functionalization, it has reduced the surface charge of CNPs from +28.4 mV to + 16.06 mV. Furthermore, subsequent drug loading and OX26 surface functionalization of CNPs have led to an increase in particle size up to 637 nm, which is unfavorable for BBB penetration. Other than that, the entrapment efficiency has increased from 7.9% for CNPs alone to 31.13% for PEGylated CNPs.

### 3.1. Physicochemical Factors Governing Chitosan-Based Interpolyelectrolyte Complexes (Stoichiometry and PH)

Stoichiometry surely plays a role in determining the physical properties of CNPs. One example is the effect of the degree of deacetylation; the higher the concentration of NaOH added to chitin, the higher the cleavage between carbon in the carbonyl group and Nitrogen in the amino group, which leads to the break of the acetamido group (–NHCOCH_3_) in chitin and release of acetate ions (CH_3_COO^−^). This eventually leads to the exposure of free amine groups (–NH_2_) on the polymer backbone, which contribute to the zeta potential of CNPs when they are protonated. Also, the greater the deacetylation degree, the smaller the CNPs are because of the broken –NHCOCH_3_ groups. As mentioned in the previous section, the experiment performed by Yang and Hon [22] has reported how 90% degree of deacetylation led to a smaller mean size of 95 nm compared to 75% DD that resulted in a mean particle size of 176 nm.

Moreover, the preparation settings of CNPs can also have an impact on their sizes. For example, Liu et al. [23] have reported that the ratio of Chitosan to TPP 6:1 has yielded CNPs in a size range of 300–400 nm. Thakur and Taranjit [55] have observed a relationship between chitosan/sTPP ratio and CNPs size, as the ratio increases (less sTPP (sodium tripolyphosphate) introduced during ionic gelation), the larger is the size of CNPs, and vice versa (lower chitosan/sTPP ratio or increased sTPP compared to chitosan yields smaller size CNPs). These changes in size occur due to changes in electrostatic crosslinking and chain interactions.

The pH of the solution during CNPs preparation is another factor that influences the interactions between polyelectrolytes, which in turn affect the physical properties of CNPs. Thakur and Taranjit [55] cite a study reporting that electrostatic interactions between polyanions and chitosan during the formation of CNPs mainly depend on the pH of the mixing solution, as they occur only in a certain pH range corresponding to the anion’s natural characteristic. TPP, for example, requires a pH range of 1.9–7.5 in order to interact with chitosan, for CNPs-sulfate it is 1.0–7.5, and it is 4.5–7.5 for CNPs-citrate. In acidic pH, the amine groups of chitosan become protonated. Protonation of the amine groups is essential as it allows for the interaction with negatively charged polyanions. Not only that, pH also affects particle size and zeta potential. The same study mentions that the CNPs particle size was observed to be growing rapidly as the pH increases from 1 to 3.5, and then it slowly decreases when the pH is increased from 3.5 to 5.5. The zeta potential, on the other hand, has increased from pH 1 to 4, then decreased gradually between pH 4 and 5.5. This behavior happens because in acidic conditions, the chitosan amine groups become protonated, resulting in higher electrostatic repulsion and a stretched molecular conformation. At a pH range between 4.5 and 6, CNPs tend to decrease in particle size and zeta potential, which in turn results in instability that can be attributed to the shielding of the chitosan positive surface charge either due to molecular structure reorganization or the adsorption of other negatively charged ions at low pH.

### 3.2. Key Methods for CNP Synthesis

CNPs can be prepared by multiple methods. The main methods are ionic gelation, complex coacervation, and self-assembly. Each method provides different characteristics of size and surface charge. Many studies used CNPs mostly by intranasal delivery because of chitosan’s mucoadhesion and positive charge for nose-to-brain transport [12].

#### 3.2.1. Ionic Gelation

Ionic gelation is the most commonly used method to make CNPs. It includes dropping a multivalent anion (mostly TPP) into an acidic chitosan solution. The negative phosphate groups of TPP crosslink with the positively charged NH_3_^+^ groups of protonated chitosan by ionic interactions. This forms a colloidal suspension of nanogels without chemical crosslinkers. The particle size and surface charge depend on many factors, including CS:TPP ratio, molecular weight, and degree of deacetylation, solution pH, and ionic strength [56]. For example, increasing the TPP content or ionic strength provides a more compact and smaller particle [57]. In one study, highly crosslinked CS/TPP nanogels are as small as 40 nm with 51 mV zeta, and particles with additional crosslinkers reached 257 nm [56]. More commonly, ionic gelation CNPs are 100–300 nm. For example, thiolated CNPs loaded with selegiline by ionic gelation were 215 nm with 17 mV [12].

Ionic gelation is simple because it uses only aqueous solutions with no organic solvents or heat. It is reproducible and can be scaled easily (stirring at room temperature). It allows encapsulation of small or ionic drugs. They can be negatively charged drugs or nucleotides, which can even replace TPP as the crosslinker [58]. The resulting NPs are biocompatible and biodegradable. Additionally, crosslink density can be adjusted because no covalent bonds are formed [59].

A limitation of using ionic gelation is that ionic crosslinks are salt sensitive. In physiological media (e.g., isotonic saline), CS/TPP NPs tend to swell, dissociate, or aggregate due to competing ions. Stability can be improved by adding salt or metal ions (e.g., Fe^3+^) during preparation. However, this makes it complex. Another limitation is the limited encapsulation of high MW or hydrophobic drugs since no covalent network is formed; large proteins or hydrophobic molecules can be hard to trap unless more modifications are made. Finally, it is difficult to control the size precisely, as small variations in mixing or concentration can provide different sizes [60,61].

In brain delivery applications, ionic gelation nanoparticles are used for nasal delivery. For example, thiolated chitosan nanoparticles prepared by ionic gelation enhanced mucoadhesion and improved nose-to-brain uptake of drugs such as selegiline, which is an antidepressant, and galantamine, which is an Alzheimer’s drug. In these cases, the CNPs were on the order of 150 to 250 nm, providing high encapsulation efficiency (50–80%) and positive zeta potential from 15 to 50 mV [12].

#### 3.2.2. Complex Coacervation

Complex coacervation refers to making CNPs by mixing chitosan with an oppositely charged polymer or molecule to create spontaneous phase separation into polymer-rich nanoparticles. This means combining Chitosan with a polyanion such as alginate, hyaluronic acid, carrageenan, dextran sulfate, DNA/RNA, etc. The two polymers interpenetrate by electrostatic and hydrogen bonds to form polyelectrolyte complexes [56]. No synthetic crosslinker is needed, and the negative charges on the second polymer act such as TPP.

The particles formed by complex coacervation are in the hundreds of nanometers range. For example, chitosan DNA polyplexes prepared by coacervation were about 260 nm in diameter [28]. Chitosan–alginate nanogels are a few hundred nm. The surface charge can be low or reversed. If the mixing ratio is balanced (charge ratio ~1:1), the nanoparticles can be neutral and even aggregate. If one polymer is in excess, the nanoparticles carry the excess charge [62]. In one study, bare chitosan NPs were 28.8 mV, but after complexing with DNA, the particles were only 10.6 mV [28].

In practice, chitosan coacervation uses specific pairs of polymers. For example, chitosan alginate or chitosan–carrageenan nanoparticles are formed by mixing solutions at acidic pH. The charge ratio (CS NH_3_^+^ groups to polyanion COO^−^/SO_3_^−^ groups) is an important parameter. Away from neutrality, the nanoparticles carry the excess charge, but at 1:1 stoichiometry, they tend to aggregate [62]. A chitosan–alginate coacervate used for nasal peptide delivery had two size populations (240 nm and 286 nm) with moderately positive zeta [12]. More generally, coacervate nanoparticles can have the same size range as ionic-gel nanoparticles, but their surface charge and stability are more variable.

Coacervation is very versatile. It works at physiological pH for HA, alginate, etc., in addition to water-only conditions. It can encapsulate charged biomolecules such as genes, proteins, and peptides without denaturing them. Also, many polyanions used are biocompatible and biodegradable, such as alginate, HA, and dextran sulfate. Because no covalent bonds are formed, the complexes can exchange ions or degrade in tissue, allowing controlled release. This method has been studied for gene delivery to the brain. For example, CS–siRNA or CS–DNA NPs made by coacervation have shown efficient transfection of brain cells when delivered intranasally [12].

However, similar to ionic gelation, polyelectrolyte complexes are sensitive to charge balance and ionic strength. If the chitosan and polyanion are mixed in a stoichiometric ratio, neutralization can lead to aggregation into large particles [63]. Therefore, the CS: polyanion ratio must be carefully tuned. The pH and ionic strength must also be controlled, as high salt can screen the interactions and destabilize the complex [64]. In practice, coacervation often provides a broader size distribution and larger particles than ionic gelation. Encapsulation is limited to charged actives (or drugs conjugated to polyanions); truly hydrophobic drugs do not load well unless additional modifications are made.

#### 3.2.3. Polymer–Drug Self-Assembly

Self-assembled chitosan nanoparticles form when the polymer and drug organize without added crosslinkers. This can happen in two main ways. Amphiphilic chitosan derivatives spontaneously form particles similar to a micelle in water, or the drug itself drives the assembly. In the first case, chitosan is chemically modified by grafting hydrophobic groups. The amphiphile self-assembles above a critical aggregation concentration, entrapping hydrophobic drugs in its core. In the second case, a hydrophobic drug or polymer drug conjugate can cause the chitosan to collapse into nanoparticles [62].

Self-assembled CNPs are usually 100–300 nm in size. For example, glycol chitosan modified with 5β-cholanic acid formed stable particles around 230 nm in diameter, with suitable loading of an RGD peptide [65]. In another study, similar amphiphilic CS NPs (with doxorubicin) averaged 284 nm (±5 nm) [66]. Chitosan–phospholipid hybrid nanoparticles were reported below 280 nm with a high positive zeta (+40 mV) [67]. Self-assembly provides nanoparticles without any added crosslinker or precipitant. Encapsulation of hydrophobic drugs can be efficient. In the chitosan and cholanic acid example, loading efficiencies exceeded 85% [65]. Table 3 below shows a summary of major chitosan nanoparticle synthesis methods (ionic gelation, complex coacervation, and self-assembly), their mechanisms, particle properties, and advantages and limitations in brain-targeted drug delivery.

According to Table 3, all methods produce positive-surface particles because of chitosan’s amines unless they are overcoated or neutralized. In brain-targeting studies, sizes around 100–300 nm are common for effective nasal to brain or intravenous delivery.

### 3.3. CNPs Shape, Packing Density of Polymer Chains, and Elastic Modulus

Chitosan nanoparticles made by ionic gelation are mostly quasi-spherical in shape. Spherical particles can pack more densely in suspension and have uniform distribution, which leads to very consistent drug release [68]. Shape modification can modulate cellular uptake. For example, in one study, antibody-coated rod-like particles showed 1.6 times higher targeted uptake by HER2^+^ cancer cells than spherical ones [69]. Moreover, in a human BBB flow model, spherical particles associated more strongly with the brain endothelium, while rod-shaped particles had higher transport per associated particle [70]. Almost all CNPs are spherical, but if they are elongated, such as rod or disk-shaped designs, they can change how CNPs bind or enter cells and how efficiently they cross the BBB [69,70].

Packing density is how tightly the polymer chains are crosslinked inside the nanoparticle. A denser polymer network, such as using more crosslinker or higher polymer concentration, can slow drug diffusion and degrade more slowly. For example, increasing the amount of TPP crosslinker in chitosan–TPP nanoparticles produced a more compact network that maintained protein release and lowered the particle degradation rate [71]. Therefore, higher crosslink density locks in the drug longer. One study showed that adding sodium TPP provided strong crosslinks into the chitosan matrix, which stabilized the particle and controlled or slowed drug release [68]. Therefore, changing the packing density of chitosan chains affects release kinetics. Very dense CNPs give slow, extended release for the drug [68,71].

The stiffness of the nanoparticle also affects cellular uptake and BBB transport. Stiff particles adhere more to cell surfaces, while very soft particles can navigate tight spaces differently. In a human BBB microfluidic study [70], stiff CNPs showed higher endothelial association than soft CNPs. However, when they are normalized for how many particles were bound, soft and hard particles had similar BBB penetration. In that work, 200 nm stiff polystyrene spheres bound strongly to endothelium, while similar-sized soft hydrogel spheres bound less but still crossed the barrier almost as efficiently once association was accounted for. However, CNPs are relatively soft because of the hydrogel matrix compared to rigid plastics, which can create some deformation during uptake. Because stiffer matrices also slow down drug diffusion, tuning chitosan stiffness can also be applied. Softer chitosan gels can give faster uptake but with faster release, while stiffer and more crosslinked gels resist uptake but release the drug more slowly. Overall, the balance of stiffness affects the rate and route of uptake into brain cells and the BBB [70].

## 4. Therapeutic Applications of CNPs

Many in vitro and in vivo studies have been conducted to test the therapeutic potential of CNPs for various brain disorders and transition to clinical trials. The most common brain disorders, such as neuro-degenerative diseases, brain tumors/gliomas, and ischemic stroke, are chosen to be discussed in this paper.

### 4.1. Treatment of Neuro-Degenerative Diseases: Alzheimer’s, Parkinson’s, and Huntington’s Disease

**Figure 5:**
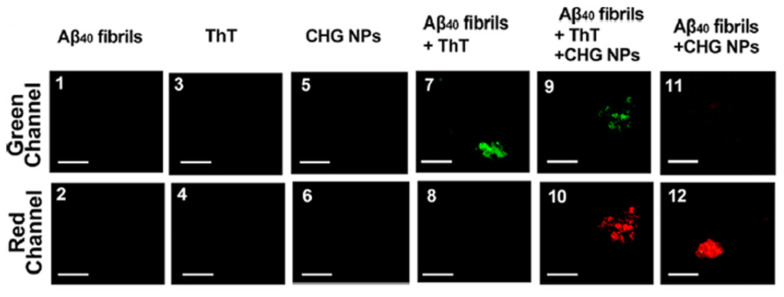
Microscopy of Aβ fibrils stained with both ThT (green) and CHG-NPs (red) co-localized on the same fibrils [72].

The key molecular indicators of Alzheimer’s disease (AD) (shown in Figure 3) are the aggregation or buildup of amyloid-beta plaques in the brain and the hyperphosphorylation of tau proteins, which are microtubule-associated proteins involved in stabilizing neurons, leading to neurofibrillary tangles or NFTs. Many studies have been conducted that included different approaches to target these pathological molecules for treating Alzheimer’s disease. In a study made by Wang et al. [72], they have successfully developed chitosan nanoparticles loaded with hyaluronic acid and cross-linked with glutaraldehyde that were found to be able to detect and inhibit amyloid-beta fibrillization in vitro and in vivo. The chitosan-hyaluronic acid nanoparticles (CHG NPs) in the study have shown high sensitivity and selectivity for amyloid-beta plaques, as the fluorescence imaging has shown an intense red color following the interaction of these nanoparticles and amyloid-beta oligomers and fibrils. The inhibition of amyloid fibrils was almost completely achieved, which demonstrates the high significance of CHG NPs as therapeutic/diagnostic agents for treating AD. The detection efficiency of 7 μg/mL CHG NPs was compared to that of 10 μM Thioflavin (ThT), ThT is a gold-standard fluorescent probe for detecting amyloid fibrils in Alzheimer’s research. Thioflavin fluorescence emission, on the other hand, is visualized through the green channel (green emissions). The CHG NPs were able to detect amyloid fibrils earlier compared to ThT. Figure 5 below demonstrates the detection capability of CHG NPs, which is comparable to that of ThT [72].

Similarly to Wang et al. [72] study, Aβ fibril detection was successful in an experiment performed by Noah and Ndangili [73], where they developed chitosan nanoparticles coated with PGLA and conjugated with a novel anti-amyloid-beta antibody; the results have shown an enhanced targeting of the amyloid-beta aggregates and their inhibition. Al-sarayra et al. [74] have designed a nanocomposite of chitosan and gold with optimized concentrations for an efficient particle size of 39.2 nm and donepezil drug loading of 35.5%. This nanocomposite has demonstrated high potential for AD treatment as it showed a desired controlled release of donepezil with a 75% release after 1440 min. In other papers [75,76,77], chitosan-based nanoparticles have shown excellent encapsulation efficiency, reduced systemic side effects or toxicity, and high brain uptake of the drug.

**Figure 6:**
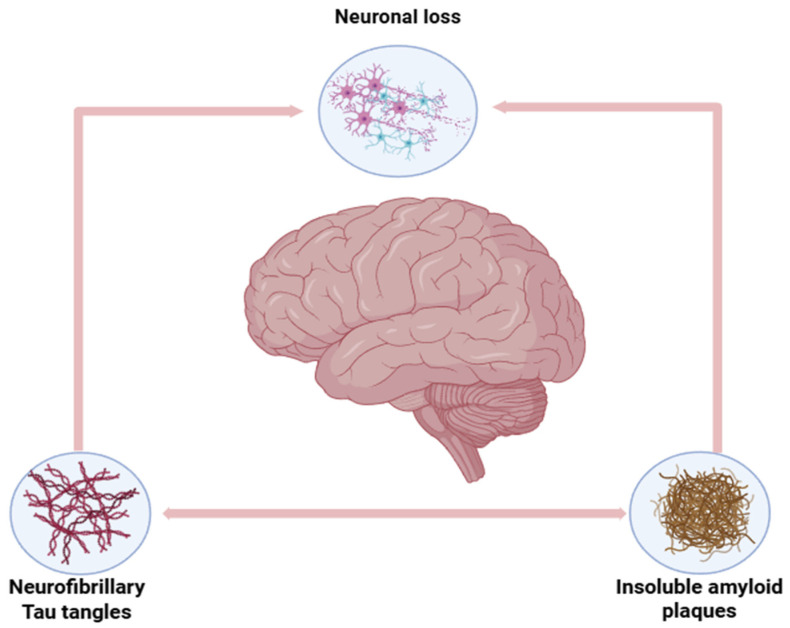
Pathological markers of Alzheimer’s Disease: amyloid-beta and NFTs.

In a study performed by Shafqat et al. [78], they have tested betanin-encapsulated chitosan nanoparticles, they have showing robust antioxidant and anti-inflammatory effects. Antioxidants such as betanin are essential for their incorporation in chitosan nanoparticle DDS for Alzheimer’s as there is an over production of what are called the “reactive oxygen species” or ROS that occur due to the amyloid beta aggregation; the oxidative stress also in turn promotes and amplifies amyloid-beta aggregation and tau hyperphosphorylation (NFTs), creating a vicious cycle that contribute to more neuro-degeneration, memory deficits and synaptic loss [79,80]. Figure 6 below shows the pathological markers of Alzheimer’s Disease: amyloid-beta and NFTs.

Another neuro-degenerative disease, such as Parkinson’s Disease (PD), is characterized by the aggregation of misfolded alpha-synuclein protein in the brain that leads to the formation of Lewy bodies, which eventually leads to the degeneration of dopaminergic regions in the substantia nigra (a critical brain region for the production of dopamine [81]). Similarly to AD, PD is also accompanied by oxidative stress [82]. The current approaches followed for treatment of PD focus on management of the motor symptoms by using levodopa, monoamine oxidase-B inhibitors, and dopamine agonists. However, the emerging approaches that use chitosan nanoparticles aim to target the underlying pathology of PD, such as minimizing alpha-synuclein and enhancing mitochondrial function [16]. In a study performed by Martínez et al. [83], a novel carrier system of chitosan-coated solid lipid with an average size of 250 nm was developed for delivering dopamine across the BBB, and it was able to pass across the endothelial cells in vitro, which makes these nanoparticles have potential for enhancing dopamine bioavailability in the brain when used in clinical treatment of PD.

Saha et al. [84] developed a lecithin–chitosan nanoparticle for loading rotigotine, which is a dopamine agonist that was already used to treat PD and restless leg syndrome through oral administration; however, rotigotine suffers from oral bioavailability and first-pass metabolism. The developed rotigotine-loaded lecithin chitosan nanoparticles have demonstrated improved brain drug delivery (through the nasal route) and targeting efficiency. In another promising study performed by Sardoiwala et al. [85], they assessed the neuroprotective potential of chitosan nanoparticles loaded with PP2A activator FTY720 that minimizes the phosphorylated alpha-synuclein. The results have shown a reduction in the PD marker pSer129 alpha-Synuclein, and there was an observed interaction between PP2A and the protein enhancer of zeste homolog 2 that led to degradation of aggregated alpha-synuclein. This is analogous to the detection and inhibition of Aβ fibrils in AD.

Multiple studies also focused on treating Huntington’s disease (HD), a neuro-degenerative disease that occurs as a result of an autosomal Deoxyribonucleic Acid (DNA) dominant inheritance pattern in the form of repeated CAG trinucleotide in the huntingtin “HTT” gene. This later leads to the synthesis of mutant huntingtin protein (mHTT) that has extended repeats of glutamine (an amino acid) [16]. In a study performed by Fihukra et al. [86], they developed hybrid-chitosan-based nanocarriers loaded with small interfering RNAs (small interfering ribonucleic acid (siRNA)) that can reduce the mHTT levels and inflammation. The result of testing these nanocarriers on the stem cells of a mouse with HD showed an effective reduction in mHTT and reduced inflammation. Wahyuningtyas et al. [87] approach was to prevent the formation of harmful amyloid fibrils by the polyglutamine-rich peptides or mHTTs by the use of amphiphilic peptides that self-assemble into vesicles, and they were also conjugated to chitosan nanoparticles. This nanocomposite was able to penetrate the cells, inhibit mHTT aggregation, and reduce its toxicity.

### 4.2. Treatment of Brain Cancer

As mentioned in the introduction, there are certain surface modifications that can be implemented on the chitosan nanoparticles for specific targeting of brain tumors, such as modifying with tumor-targeting peptides, such as transferrin and chlorotoxin, to target brain tumors with over-expression of TfR or Matrix Metalloproteinase-2 (MMP-2). Nevertheless, there are other brain tumors that show over-expression of certain receptors, such as folate receptors, on their outer surface, which allows for selective targeting by modifying nanoparticles with folate [88]. These modifications enhance the delivery of the therapeutic drugs that have shown effective cytotoxicity to cancer cells, such as methotrexate (MTX), sorafenib, doxorubicin, and paclitaxel (SF) in many studies [89]. In a study conducted by Ruman et al. [90], the developed folate-coated chitosan nanoparticles loading sorafenib drug have exhibited an enhanced delivery to human hepatocellular carcinoma and colorectal adenocarcinoma cell lines. They demonstrated good release in PBS solution at pH 4.8, superior anti-cancer activity over free sorafenib, and no damage to normal cells. The folate-coated CNPs loading sorafenib (SF-CS-FA NPs) anti-cancer effects were compared to those of chitosan, pristine sorafenib, and Chitosan-sorafenib. While chitosan alone was not effective, SF-CS and SF-CS-FA nanoparticles increase cytotoxicity against cancer cells compared with free sorafenib. Due to SF-CS-FA targeting efficiency and increased cellular uptake, it has shown the lowest IC50 (concentration required to reduce cancer cells’ viability by 50%) in both cell lines.

In another study performed by Gabold et al. [91], transferrin was attached to the surface of chitosan nanoparticles as targeting ligands for the TfR on the surface of human glioblastoma cells (RPMI 2650 and U87 cells) in vitro. As Ruman et al. studied, they have also demonstrated enhanced targeting of cancer cells and exhibited increased cellular uptake. Other studies discussed gene therapy where chitosan nanoparticles act as non-viral vectors that carry some genetic material to be introduced to the cancer cells (transfection) for a genetic modification to occur by exerting a specific action. Chitosan nanoparticles offer a safer alternative than the viral-mediated drug delivery systems due to their non-toxic nature and capacity for protecting nucleic acids during the formation of complexes with genetic material; they also overcome the challenge of BBB penetration [92]. Khan et al. [28] conducted in vitro studies for chitosan nanoparticles gene delivery that showed higher cell viability of 85% in U87 cells compared to 72% using other transfection agents (other than chitosan). In addition, chitosan nanoparticles have demonstrated greater transfection efficiency (20.56% vs. 17.79%).

### 4.3. Treatment of Stroke

An ischemic stroke occurs when a blood vessel is blocked, which leads to a lack of blood supply to brain tissue [93]. This type of stroke accounts for 60 to 85% cases worldwide [94]. The cerebral ischemia–reperfusion injury (CIRI) is the damage that occurs after the blood supply returns to the brain after a period of ischemia; this leads to inflammation and oxidative damage due to an increase in inflammatory factors and reactive oxygen species (ROS), resulting in neuronal death and neurological dysfunction. Thus, anti-inflammatory agents can be utilized to eliminate the damage of ischemic brain tissue by removing inflammatory factors and repairing damaged tissue. Furthermore, anti-oxidative agents (as in AD treatment) can be delivered in encapsulation by chitosan nanoparticles to promote a neuronal cell defense against toxic ROS. In a study performed by Zhao et al. [95], they have used gallic acid-loaded chitosan nanoparticles that are coated with O-carboxymethyl for specific targeting of ischemic regions. The resulting nanoparticles showed a smooth spherical morphology with an average diameter of 173 ± 18 nm and a positive zeta potential of +21.3 ± 2.2 mV, indicating good colloidal stability. The encapsulation efficiency was 83.5 ± 6.0%, and the drug loading capacity reached 30.0 ± 6.3%, confirming high loading efficiency and favorable physicochemical characteristics for brain-targeted drug delivery. These nanoparticles were able to reduce the levels of pro-inflammatory cytokines such as TNF-alpha and IL-1Beta. Furthermore, they improved the activity of antioxidant enzymes, which contributed to the reduction in oxidative stress. In another study conducted by Nagareddy et al. [96], they produced chitosan NPs that are coated with bilirubin for targeting acute ischemic stroke lesions; these NPs are loaded with atorvastatin, which produces anti-oxidative effects. They were tested in animal models and have demonstrated an inhibiting effect on the impact of ROS, which reduced the oxidative stress in addition to decreasing the inflammatory cytokines IL-1beta and IL-6. This eventually led to improvements in motor deficits and a reduction in infarct volumes. Table 4 shows the chitosan nanoparticles’ applications for different brain disorders.

Notice that not all studies show that nanoparticles are modified with a ligand for specific targeting, as the drug used for reduction in certain molecules, such as Amyloid beta in Alzheimer’s or alpha-synuclein in Parkinson’s, is safe for the surrounding cells. However, it is a different case for the tumor cells that require cytotoxic drugs for killing those cells only; a non-specific tumor drug release can lead to the harm of surrounding healthy neuronal cells in the brain.

Overall, this section addressed the cytotoxicity of chitosan nanoparticles, which is important for potential clinical applications. The complex nature of CNP-induced toxicity is discussed with a focus on how variables such as nanoparticle concentration, size, surface charge, and the type of cell line can all alter cytotoxic outcomes. The findings show the importance of carefully optimizing these parameters to reduce toxicity and optimize the safe use of CNPs in brain drug delivery.

## 5. In Vitro/in Vivo Studies for Biocompatibility and Cytotoxicity Characterization of CNPs

**Figure 7:**
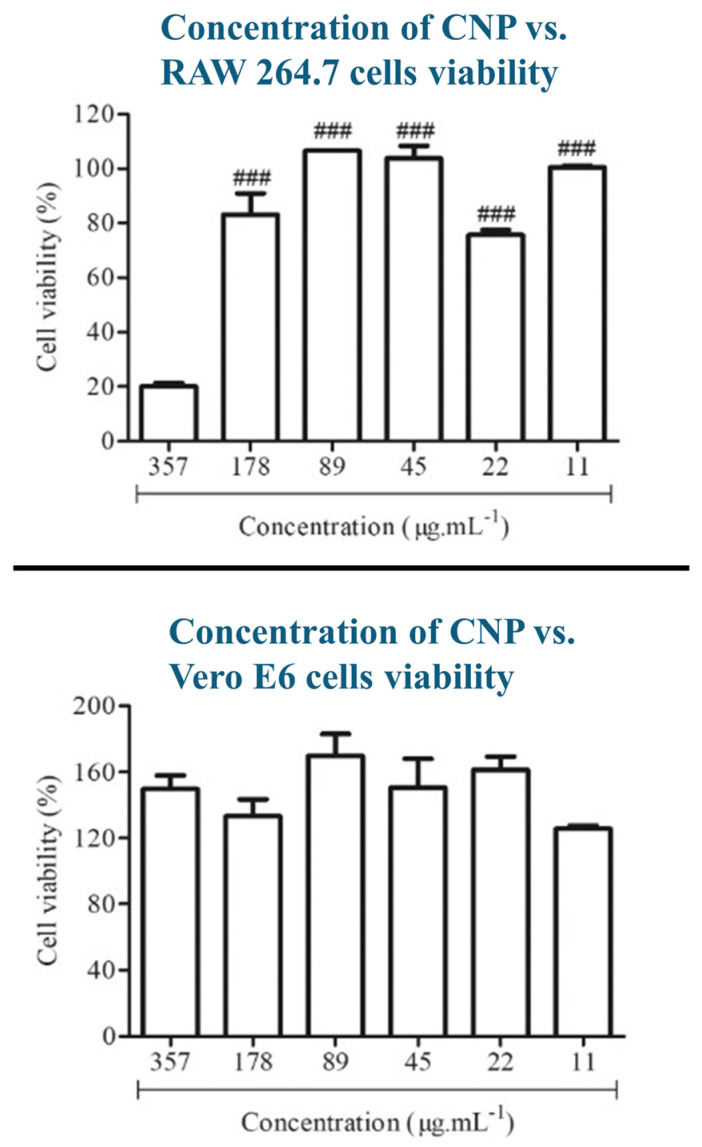
(Concentration increases from right to left) High cytotoxicity was shown at the highest concentration tested (357 ug/mL) for RAW 264.7 cells (**up**), Vero E6 cells continued to grow normally (**down**). These notations ### are for statistical analysis. They represent the differences in the mean values with a *p* < 0.001 as explained in the original work [98].

Although chitosan nanoparticles offer many advantageous properties that facilitate drug delivery (Muco-adhesive Properties, for example) to the brain for various brain disorders. Biocompatibility and possible cytotoxic effects should be considered by examining the characteristics of CNPs that can elevate their cytotoxicity through multiple in vitro and in vivo tests. The factors that affect the choice of cytotoxicity methods are exposure duration, amount, and frequency of exposure to chitosan nanoparticles, in addition to the type of exposed tissues and results from previous toxicity studies. It is necessary that in vivo or animal testing should be reduced as much as possible and replaced with in vitro studies for ethical considerations; thus, most of the studies found and included in this section are in vitro [97]. In a study conducted by Torres–Rego et al. [98], chitosan nanoparticles were incubated with vero E6 (derived from African green monkeys) and RAW 264.7 (derived from murine mice) cells to assess their cytotoxicity using the MTT assay. The CNPs were prepared through ionic gelation with TPP, and they have a mean size of 145 nm, a mean zeta potential of +21.21, and a mean PDI of 0.28. The chitosan nanoparticles have shown an excellent biocompatibility in both cell types; however, the viability of RAW 264.7 cells has drastically dropped at the highest chitosan nanoparticle concentration tested, which was 357 ug/mL, while the Vero E6 cells were not affected and they maintained a high viability, as shown in Figure 7. This shows how different cell types exhibit different cytotoxic levels at certain nanoparticle concentrations, and that higher concentrations can increase cytotoxicity.

Zoe et al. [99] conducted an in vivo study where TPP chitosan nanoparticles were incubated with Zebrafish embryos; smaller nanoparticles showed a higher toxicity than larger nanoparticles. This confirms how the size of chitosan nanoparticles can affect their cytotoxicity [99]. Ding et al. [100] have implemented two in vitro assays (CCK-8 and hemolysis assays) to measure the cytotoxicity of chitosan nanoparticles before and after thiolation (cross-linking thiol groups to the nanoparticle surface) against DC2.4 cells, RAW264.7 cells, and chicken erythrocytes. The authors described an observed reduction in cytotoxicity after thiolation of chitosan nanoparticles that reduced the surface charge compared to the higher cytotoxicity of unmodified chitosan nanoparticles that had a higher surface charge. This result is confirmed by another paper performed by Wani et al. [101] that mentions high surface charge of nanoparticles as a factor of cytotoxicity, alongside smaller sizes of nanoparticles [102]. An in vivo study [103] has been performed on rats that were exposed to chitosan nanoparticles for seven days to study the effect of exposure duration on cytotoxicity; this resulted in neuronal apoptosis and necrosis (death of cells), which accumulated in the rats’ frontal cortex and cerebellum. The neuronal cells have decreased in the viability of neural cells in a dose-dependent manner. This indicates how long exposure durations affect the cytotoxicity of chitosan nanoparticles.

Considering these studies, the following conclusions can be drawn about the factors and characteristics related to chitosan cytotoxicity: the higher the concentration/dose of CNPs, the higher the cytotoxicity. The smaller the particle size, the higher the cytotoxicity compared to larger sizes. The higher the surface charge or zeta potential, the higher the cytotoxicity. Longer exposure duration of cells to CNPs elevates the cytotoxicity. Different cell lines have different responses as they can show either high or low cytotoxicity for a certain concentration.

The higher concentrations of chitosan nanoparticles lead to increased cytotoxicity because of their positively charged surface that interacts with negatively charged regions on the cell membrane, which is desired because it enhances the uptake of the nanoparticles into the cell. However, higher concentrations can damage the cell membrane and cause leakage of intracellular substances, which kills these cells [100]. This, by extension, means that the higher the surface positive charge density that can come from a high degree of deacytelation, the more enhanced the interaction points between nanoparticles and cells, which results in increased cytotoxicity [101]. However, a lot of studies show that a smaller nanoparticle size contributes more to the cytotoxicity than the surface charge [97]. Smaller nanoparticle sizes have a higher surface area to volume ratio, which enhances their interaction with the cell membrane, which eventually leads to their structural damage. Additionally, because of their increased Brownian motion, smaller nanoparticles are more stable in fluids and accumulate more on cell surfaces. This raises the cytotoxic effects by increasing cellular uptake and interaction [102]. Prolonged exposure can lead to the aggregation of nanoparticles within cells, which may overwhelm biological systems and cause increasing cytotoxic effects [104].

## 6. Challenges and Future Perspectives

Chitosan is found to have a broad range of biomedical applications; however, these applications are still limited due to challenges such as poor solubility and mechanical properties. CNPs with no modifications face difficulty in transporting drugs effectively all the way to the brain because of the alkalinity of the delivery route environment (BBB and cerebrospinal fluid). Moreover, the low mechanical strength of chitosan represents a greater challenge for its application in treatments involving neuro-regeneration, where faster neural atrophy takes place prior to achieving therapeutic effects. These limitations of unmodified chitosan were addressed by many studies that developed novel surface modifications for better physicochemical characteristics. However, this can increase the nanoparticles’ size to an extent that can prevent them from passing through the BBB, and these surface modifications still do not completely address the issue of cytotoxicity. Therefore, more studies should be implemented towards the possible physical aspects, such as those discussed in Section 5 (Molecular size, Surface charge, Concentration, and exposure duration), to standardize the optimal parameters that yield the lowest cytotoxic effects possible. The variation in cytotoxic effects among different cell lines should also be considered in these studies. Future efforts should all be focused on novel modifications of CNPs because many of them have already addressed many challenges associated with their use in previous studies. Moreover, more comprehensive research should be conducted on the selectivity of these drug delivery systems, in vitro and in vivo toxicity, safety problems linked to chitosan-based biomaterials, and the complexities of their manufacturing processes [7].

## 7. Conclusions

This paper discussed Chitosan-based nanoparticles for brain drug delivery. As seen in the literature, the major challenge in brain drug delivery is the blood–brain barrier, which restricts the passage of multiple drugs that can treat multiple neurological disorders. CNPs carriers can be used in drug delivery because of their ability to encapsulate drugs, protect them from degradation, and deliver them in a controlled manner. Their cationic properties allow them to interact with the negatively charged components of the BBB, which improves adsorptive-mediated endocytosis and permeability. Moreover, surface modifications, such as PEGylation, ligand conjugation, and hybridization with other polymers (e.g., PLGA), can improve their stability, targeting efficiency, and therapeutic results. Drug-loaded CNPs can be used to treat multiple brain disorders, including neuro-degenerative diseases, brain tumors, and ischemic strokes. In Alzheimer’s disease for example, CNPs are effective in targeting pathological markers such as amyloid-beta plaques and tau proteins through ligand-modified CNPs. Parkinson’s disease treatment includes the use of dopamine-loaded CNPs and carriers targeting misfolded alpha-synuclein proteins. For ischemic strokes, CNPs with anti-inflammatory and antioxidant drugs can reduce neuronal damage. Intranasal delivery was shown to be the most effective route for administering CNPs to the brain, as it allows for bypassing first-pass metabolism and provides direct access to the central nervous system. The mucoadhesive properties of chitosan can enhance residence time in the nasal cavity, which improves drug absorption and BBB penetration. Size optimization also determines transport efficiency across the BBB. Suitable CNP size for brain drug delivery ranges between 30 and 300 nm. Cytotoxicity is influenced by factors such as nanoparticle size, surface charge, and concentration. Smaller nanoparticles have better BBB penetration, but they have higher toxicity because of the increased surface area and interaction with cellular membranes. Prolonged exposure to high concentrations of CNPs can lead to dose-dependent toxicity.

## Figures and Tables

**Table 1:** Comparison between intranasal, oral, and intravenous routes.

Route	Description/Mechanism	Advantages	Limitations	Ref
Oral	The drug is administered through the gastrointestinal tract and absorbed into the systemic circulation before reaching the brain via the bloodstream.	Convenient and non-invasive for patients. Suitable for chronic administration.	Undergoes first-pass metabolism, which reduces the amount of drug reaching the brain. Low brain targeting efficiency	[11]
Intranasal	The drug is administered through the nasal cavity and absorbed into the brain through the olfactory pathway.	Avoids first-pass metabolism. Rapid drug onset and direct brain targeting. Chitosan provides mucoadhesion, prolonging. residence time and improving absorption.	Limited drug volume per dose. Mucociliary clearance can lower the drug retention. Larger particle sizes (>300 nm) can hinder mucosal transport and uptake.	[13,14]
Intravenous	Direct injection of the drug into the systemic circulation for rapid distribution to body tissues.	Rapid systemic distribution provides controlled dosing.	Possible systemic side effects. Requires clinical administration.	[12]

**Table 2:** Comparative analysis of chitosan-based nanoparticles for brain drug delivery.

Nanoparticle Composition/Encapsulated Drug	Size (nm)	Zeta Potential (mV)	Entrapment Efficiency (%)	Drug Delivery Route	Key Findings	Ref.
PLGA with fragmented chitosan (CS) coating	211.9 ± 14.04	+7.1 ± 2.3 38	34.37	-	Enhanced drug delivery across the BBB, 17.18% drug loading; coated PLGA NPs for neuronal cells	[41]
PLGA and oligomeric chitosan (OCS) with CTX conjugation	213–875 (Optimal 258)	+37 (optimal)	75.69 to 93.23 (Optimal 88%)	Nasal	Nasal co-delivery of CHC and CTX to the brain; high positive charge and stability optimized by emulsification	[42]
Chitosan nanoparticles for VIN delivery	130.6 ± 8.38	+40.81 ± 0.11	-	Intranasal	High brain delivery efficacy with intranasal administration; enhanced stability due to high zeta potential	[43]
Chitosan nanoparticles for lomustine	190 to 255	-	77.12 to 88.74	-	Diffusion-controlled release over 8 h; optimized by Box- Behnken design	[44]
Glycol chitosan-coated lipid carrier (GC-ANLC)	184.2 ± 5.59	+18.83 ± 1.18	83.52 ± 2.59	Intranasal	2.3- to 4-fold higher brain bioavailability in rats; high biocompatibility with nasal epithelial cells	[45]
TPGS-conjugated chitosan (TPGS-CS) micelles, TfR-targeted, loaded with docetaxel (DTX)	16.12 ± 2.2	1.11 ± 0.57	98.9	Intravenous (iv)	2.9- to 4.1-fold higher bioavailability in vivo; 97- to 248-fold increase in vitro cytotoxicity against glioma cells; effective targeting of TfR—overexpressed glioma	[46]
Chitosan nanoparticles encapsulating anti-Gal-1 siRNA	141 ± 5	+32	81 ± 3	Intranasal	Protected siRNA from degradation; enhanced nasal retention and CNS penetration; downregulated Gal-1 expression; inhibited GBM tumor progression	[47]
Tannic acid-loaded PLGA nanoparticles coated with chitosan (2% and 4%)	Uncoated: 105.7 ± 11.02; Coated: 117.2 ± 3.09	Uncoated: −22.3 ± 2.3; Coated: +21.6 ± 1.09	Uncoated: 69.31 ± 5.89; Coated: 73.94 ± 4.28 up to 74.64 ± 4.91	Intranasal	Higher brain bioavailability and therapeutic efficacy in epilepsy models; enhanced mucoadhesion and brain targeting; safe based on toxicological evaluation	[48]
Crocin nano-chitosan-coated compound (CNCC)	175 ± 5	-	85	-	Improved memory, learning, and anxiety indicators; upregulated NMDA receptor subunits and BBB tight junction proteins; more effective than intact crocin or chitosan	[49]
N-trimethyl chitosan chloride (TMC) nanoparticles loaded with anti-neuroexcitation peptide (ANEP)	255	+32	80.63	Intravenous	Enhanced brain distribution by absorption-mediated transcytosis; effectively delivered ANEP to the brain	[50]
Dual antibody-modified chitosan nanoparticles (anti-Tf and anti-B2) loaded with siRNA	235.7 ± 10.2	+22.88 ± 1.78	61.9	-	Enhanced cellular uptake and gene silencing efficiency in astrocytes; significantly improved knockdown of HIV replication compared to non-modified and single-antibody-modified nanoparticles	[51]
Chitosan-PEG-PEI copolymer nanoparticles functionalized with chlorotoxin and loaded with anti-Ape1 siRNA	48.5 ± 4.0	+13 ± 3.4	-	Intravenously through the tail vein in the mouse model	Reduced Ape1 expression and increased GBM radiosensitivity; 40% Ape1 activity reduction in tumor tissue; doubled survival extension in GBM mouse models when combined with radiotherapy	[52]
Chitosan nanospheres conjugated with PEG and anti-caspase peptide Z-DEVD- FMK, modified with OX26 monoclonal antibody using SA-biotin technique	149.73 ± 1.85	+16.06 ± 3.43	31.13 ± 1.61	Intravenous	Localized in brain tissue and outside intravascular compartment, successfully delivered Z- DEVD- FMK to brain tissue as confirmed by electron microscopy	[53]
Rutin-encapsulated-chitosan nanoparticles (RUT-CS-NPs) prepared by ionic gelation	92.28 ± 2.96	+31.04 ± 1.91	84.98 ± 4.18	Intranasal	For particle size less than 100 nm, enhanced nasal permeability (>80% in 24 h), 3-fold higher brain uptake, and increased bioavailability compared to free rutin; reduced cerebral infarction volume in cerebral ischemia models	[54]

**Table 3:** Summary of major chitosan nanoparticle synthesis methods (ionic gelation, complex coacervation, and self-assembly), their mechanisms, particle properties, and advantages and limitations in brain-targeted drug delivery.

Method	Mechanism	Size/Zeta	Advantages	Limitations	Ref.
Ionic Gelation	Mix Chitosan with multivalent anion (e.g., TPP), forming ionic crosslinks.	Size: 40–300 nm (e.g., 40 nm to 250 nm). Zeta: 20 to 50 mV typically (highly positive).	Very simple and mild (aqueous, no covalent crosslinkers); good encapsulation efficiency for small/ionic drugs; easily scaled.	Salt-sensitive (may swell/dissociate in physiological buffer); less stable if diluted; hard to load very large or hydrophobic drugs; sometimes broad size distribution.	[12,56,57,58,59,60,61]
Complex Coacervation (Polyelectrolyte Complex)	Mix CS (poly-cation) with an anionic polymer (e.g., alginate, HA, DNA) to form a polyion complex.	Size: 100–300 nm (e.g., 260 nm reported). Zeta: low positive or near neutral; can be adjusted by charge ratio.	No chemical crosslinkers; can encapsulate charged biomolecules (DNA, peptides) under mild conditions; uses biocompatible polyanions.	Highly sensitive to charge ratio—1:1 mixing can give neutral aggregates; pH/ionic strength must be controlled; larger particles; limited loading for neutral/hydrophobic drugs.	[28,62,63,64]
Self-Assembly (Polymer–Drug)	Use amphiphilic CS derivatives or polymer–drug conjugates that spontaneously assemble (e.g., hydrophobic grafts such as cholanic acid).	Size: 150–300 nm (e.g., 230 nm or 284 nm). Zeta: high positive (e.g., +30 to +50 mV) due to CS shell.	Encapsulates hydrophobic drugs well (high loading efficiency); no extraneous crosslinker needed; mild processing.	Requires polymer modification (synthesis time); assembly is sensitive to the degree of substitution; controlling size/composition can be tricky.	[65,66,67]

**Table 4:** Chitosan nanoparticles applications for different brain disorders.

Alzheimer’s Disease				
CNP Composition	Drug	Aim/Target	Findings	Ref.
Chitosan-coated PLGA nanoparticles conjugated with a novel anti-Amyloid antibody	Anti-amyloid-beta antibody	Amyloid-beta protein	Enhanced uptake at the BBB and better targeting of the Amyloid Beta proteins in vitro	[73]
Chitosan nanoparticles cross-linked with glutaraldehyde	Hyaluronic acid	Amyloid-beta (A*β*) protein	Was able to detect and inhibit amyloid-beta fibrillization in vitro and in vivo	[72]
Chitosan nanoparticles coated with gold	Donepezil	To inhibit acetylcholinesterase to slow AD progression	Shown potential for AD treatment as it showed a desired controlled release of the drug	[74]
chitosan nanoparticles (CS- RHT NPs)	Rivastigmine	To improve bioavailability and brain uptake of rivastigmine for Alzheimer’s disease treatment by intranasal delivery.	High encapsulation efficiency (85.3%) and sustained release over 24 h; Improved nasal mucosa permeability and brain targeting efficiency (355%); Direct nose-to-brain transport (71.8%) with enhanced brain deposition.	[75]
Galantamine hydrobromide—chitosan complex nanoparticles (CX-NP2)	Galantamine hydrobromide (GH)	To investigate if GH/chitosan complexation improves therapeutic potential for Alzheimer’s disease (AD) without altering pharmacological or toxicological profiles.	CX-NP2 significantly decreased brain acetylcholinesterase (AChE) protein level and activity compared to oral and nasal GH solutions; No toxicity or histopathological abnormalities were observed; Nanoparticles localized intracellularly within brain neurons, confirming their potential for intranasal AD management.	[76]
Donepezil (DPZ)-loaded nanostructured lipid carriers (NLCs) coated with chitosan (CH)	Donepezil (DPZ)	To enhance brain delivery of donepezil through the intranasal route using CH-coated NLCs.	Optimized formulation had 192.5 nm particle size, 89.85% entrapment efficiency, and 0.298 PDI; Bioavailability was 2.02-fold higher intranasally and 2.41-fold higher than intravenous delivery; showed 321.21% drug targeting efficiency and 74.55% nose-to-brain transport.	[77]
**Parkinson’s Disease**				
**CNP composition**	**Drug**	**Aim/Target**	**Findings**	**Ref.**
Chitosan nanoparticles	FTY720 (PP2A activator)	Phosphorylated-alpha-synuclein (pSer129)	Reduced levels of pSer129 alpha-synuclein, indicating neuroprotection against Parkinson’s Disease	[85]
Chitosan-coated solid lipid	Dopamine	To mitigate motor symptoms of Parkinson’s Disease	Enhancing dopamine bioavailability in the brain and reduced motor symptoms	[83]
Lecithin-chitosan nanoparticle	Dopamine-agonist rotigotine	To treat PD and restless leg syndrome	It demonstrated improved brain drug delivery (through the nasal route) and targeting efficiency	[84]
**Huntington’s Disease**				
**CNP composition**	**Drug**	**Aim/Target**	**Findings**	**Ref.**
Chitosan/amphiphilic peptides complex	Amphiphilic peptides	Mutant huntingtin protein (mHTT)	Nanocomposite was able to penetrate the cells, inhibit mHTT aggregation, and reduce their toxicity	[87]
Hybrid-chitosan-based nanocarriers	Small interfering RNA (siRNA)	To reduce the mHTT levels and inflammation in the stem cells of the mouse	It showed an effective reduction in mHTT and inflammation	[86]
**Brain Tumor**				
**CNP composition**	**Drug**	**Aim/Target**	**Findings**	**Ref.**
Folate-coated chitosan nanoparticles	Sorafenib	Human hepatocellular carcinoma and colorectal adenocarcinoma cells	Enhanced drug delivery to cancer cells, improving targeting efficiency against liver and colorectal cancers.	[90]
Transferrin-coated chitosan nanoparticles	Protein (Not specified)	Human glioblastoma cells in vitro	Enhanced targeting of cancer cells and increased cellular uptake	[91]
**Ischemic Stroke**				
	**Drug**	**Aim/Target**	**Findings**	**Ref.**
Bilirubin-coated chitosan nanoparticles	Atorvastatin	Ischemic stroke regions (anti-inflammatory and antioxidant targeting)	Reduced pro-inflammatory cytokines (TNF-*α*, *IL-*1*β*) and increased antioxidant enzyme activity, lowering oxidative stress	[96]
O-carboxymethyl-coated chitosan nanoparticles	Gallic acid	Ischemic regions	Significantly reduce the levels of pro-inflammatory cytokines and enhanced activity of antioxidant enzymes	[95]

## Data Availability

No new data were created or analyzed in this study.

## Abbreviations

The following abbreviations are used in this manuscript:

CS	Chitosan
CNPs	Chitosan-Based Nanoparticles
BBB	Blood–Brain Barrier
AD	Alzheimer’s Disease
Aβ	Amyloid-beta
CIRI	Cerebral Ischemia–Reperfusion Injury
CNS	Central Nervous System
DD	Degree of Deacetylation
DNA	Deoxyribonucleic Acid
EGFR	Epidermal Growth Factor Receptor
5-FU	5-Fluorouracil
HIF-1α	Hypoxia-Inducible Factor 1-alpha
miRNA	Micro Ribonucleic Acid
MMP	Matrix Metalloproteinase
P-gp	P-glycoprotein
PD	Parkinson’s Disease
PLA	Polylactic Acid
PLGA	Poly(lactic-co-glycolic acid)
ROS	Reactive Oxygen Species
siRNA	Small Interfering Ribonucleic Acid
sTPP	Sodium Tripolyphosphate
TfR	Transferrin Receptor
TPP	Tripolyphosphate
